# The Rich Chemistry of Ionized Methanol With Sulfur Dioxide: Pathways to Prebiotic and Elusive Species in Earth's Atmosphere and Space

**DOI:** 10.1002/chem.71375

**Published:** 2026-07-09

**Authors:** Mauro Satta, Daniele Catone, Mattea Carmen Castrovilli, Antonella Cartoni

**Affiliations:** ^1^ Department of Chemistry Institute of Nanostructured Materials‐CNR (CNR‐ ISMN) Sapienza University of Rome Rome Italy; ^2^ Istituto Di Struttura della Materia – CNR (CNR‐ISM) Rome Italy; ^3^ Department of Chemistry Sapienza University of Rome Rome Italy; ^4^ Istituto Di Struttura della Materia – CNR (CNR‐ISM) Monterotondo Italy

**Keywords:** ion chemistry, methanol activation, methanol radical cation, sulfur dioxide radical cation, synchrotron radiation

## Abstract

Sulfur dioxide (SO_2_) and methanol (CH_3_OH) are key components in both atmospheric and astrochemical environments, where ionizing radiation can initiate complex reaction networks. Here we investigate the ion chemistry of the CH_3_OH^•+^ + SO_2_ system by combining synchrotron experiments with theoretical analysis. The methanol radical cation CH_3_OH^•+^, generated at different photon energies, reacts efficiently with SO_2_, leading to an abundant distribution of ionic and neutral products. The reaction proceeds through rapid pathways involving the formation of the bound adduct [HOCH_2_O(H)SO]^•+^, which acts as a key intermediate governing the subsequent chemistry. Rearrangement and fragmentation of this ion lead to the formation of several species of chemical relevance. The formation of CH_2_O is particularly relevant because of its significant role in atmospheric oxidation processes and its importance as a prebiotic molecule detected in various interstellar environments. In addition, the formation of sulfur‐containing molecules such as HOSOH^•+^, HOSO**•**, and CH_2_OSO^•+^ is of interest because these species are involved in sulfur oxidation chemistry, aerosol precursor formation, and contribute to the chemistry of sulfur in planetary environments and in the interstellar medium. The results indicate that the reaction of CH_3_OH^•+^ with SO_2_ can efficiently generate reactive molecules capable of initiating further chemical transformations.

## Graphical abstract

The reaction between the methanol radical cation and sulfur dioxide is remarkably fast and forms several ionic and neutral products (HOSOH^+^, CH_2_OSO^+^, CH_2_OH^+^, CH_2_O, H_2_O, HSO_2_). The chemistry goes beyond simple proton or hydrogen transfer, revealing a close interaction between the two species, relevant to atmospheric and astrochemical environments where SO_2_ and CH_3_OH coexist.

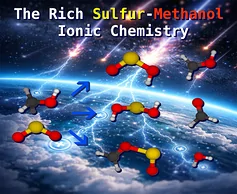

## 1 Introduction

Sulfur dioxide (SO_2_) and Methanol (CH_3_OH) are two molecules of great importance in both Earth's atmosphere and outer space. In the atmosphere, SO_2_ is mainly produced by volcanic eruptions and human activities such as fossil fuel combustion. It plays a central role in atmospheric chemistry, as it contributes to the formation of sulphate aerosols that affect climate by scattering solar radiation and influencing cloud formation in the stratosphere [1]. At the same time, it is a harmful air pollutant in the troposphere, responsible for acid rain and adverse effects on ecosystems and human health [2]. Methanol, though less abundant, is a key volatile organic compound (VOC). It participates in photochemical reactions that generate tropospheric ozone and reactive radicals, thereby regulating the oxidative capacity of the atmosphere and influencing air quality [3]. In space, these molecules provide valuable insights into planetary and interstellar processes. SO_2_ has been observed in the atmospheres of Venus, Europa, and Io, where it serves as a tracer of volcanic and geochemical activity [4]. Methanol, on the other hand, is one of the most widespread organic molecules detected in interstellar clouds and protoplanetary disks [5]. Its presence highlights the pathways through which simple molecules can evolve into more complex, prebiotic compounds essential to the origins of life [6].

Clusters formed by methanol (CH_3_OH) and sulfur dioxide (SO_2_) can also attract significant attention in both atmospheric science and astrochemistry. These small molecular aggregates, stabilized primarily by hydrogen bonding and van der Waals interactions, provide a window into the behavior of molecules at the interface between the gas phase and condensed phases. Understanding their formation and properties is essential for exploring environmental processes and the origins of prebiotic chemistry [7, 8]. Cluster formation may influence early stages of aerosol nucleation, affecting cloud condensation nuclei and climate processes [9]. In the interstellar medium, clustering could facilitate prebiotic chemistry by providing environments for the formation of complex organic molecules essential to chemical evolution [10]. The study of ionized clusters of methanol (CH_3_OH) and sulfur dioxide (SO_2_) is pivotal in understanding both atmospheric and astrochemical processes where ionizing sources are present. For instance, studies have shown that the ionization of methanol clusters leads to the formation of protonated methanol species, which can further participate in chemical reactions relevant to astrochemical processes [11, 12]. Furthermore, the study of ionized methanol–SO_2_ clusters can provide insights into the chemical processes occurring in star‐forming regions. Observations have detected emissions from methanol and SO_2_ in these regions, suggesting their involvement in the chemical evolution of the interstellar medium [13]. Sulfur dioxide (SO_2_) is also a chemical substance that SRM (solar radiation management) technology aims to use and inject into the stratosphere to counteract global warming caused by greenhouse gases [14]. In fact, SO_2_, like that released into the stratosphere by major volcanic eruptions such as the 1991 Mount Pinatubo eruption, can form H_2_SO_4_ aerosols, which partially reflect sunlight back into space instead of allowing it to reach the Earth's surface. However, there are many concerns about this technology because SO_2_ is a reactive species, both as a cationic and radical [15], towards molecules present in the stratosphere such as hydrogen, water, and methane [16, 17]. This reactivity could alter stratospheric chemistry, with effects that are still not fully understood or predicted.

CH_3_OH^•+^ radical ion through reactivity with CH_3_OH, CH_4_, and O_2_ present in the environment [18, 19, 20] can lead to prebiotic species and reactive radicals such as CH_3_• and •OOH. The latter affects ozone chemistry, increasing O_3_ in the troposphere, especially when there is a high solar radiation intensity, and decreasing O_3_ in the stratosphere [21]. The ionic species can be produced by cosmic rays, thunderstorms, lightning, and high‐voltage pipes.

Based on literature data and the above considerations, we have explored both experimentally and theoretically the reactivity of the methanol radical cation (CH_3_OH^•+^), generated by synchrotron radiation at various photon energies, with SO_2_ to investigate the formation of neutral and ionic products of interest in atmospheric and astrochemical contexts. The results reveal a complex chemistry that extends beyond simple hydrogen or proton transfer reactions, leading to the formation of abundant stable molecules in both environments, such as water and formaldehyde. Moreover, several elusive and highly reactive species are formed, including H_2_SO_2_^+^, CH_2_SO_2_^+^, CH_2_OH^+^, and HSO_2_•. These reactive species can initiate fast secondary reactions, potentially contributing to the synthesis of more complex organic molecules. This behavior highlights the role of [CH_3_OH─SO_2_]^•+^ molecular system as a possible starter of pathways for the chemical evolution from simple to more complex species in both atmospheric aerosols and interstellar environments where mixture of CH_3_OH and SO_2_ in ice and gas phase can be bombarded by cosmic radiations and produce elusive species responsible of the abundant and still not completely known sulfur and methanol chemistry [22, 23]. For the sake of clarity, the point •, indicating the radical nature of the cation, will be omitted. Moreover, the mass‐to‐charge ratio *m/z* will sometimes be substituted by number^+^, for example: *m/z* = 32 with 32^+^.

## 2 Methods

### 2.1 Synchrotron Experiments

The experiments were carried out at the CiPo (Circular Polarization) beamline of the ELETTRA synchrotron facility, employing the setup and procedures described in our previous studies and partially reported here [24, 25, 26, 27]. Radiation was generated by an electromagnetic elliptical undulator/wiggler and subsequently monochromatized using a normal incidence monochromator (NIM) operating in the vacuum ultraviolet (VUV) photon energy range of 8–40 eV. For the present experiments, an aluminum grating restricted the working range to 8–17 eV, providing a resolving power of approximately 1000. Photon energy calibration was achieved by referencing the autoionization features of the 3p spin–orbit components in the total photoionization spectrum of argon. To suppress higher‐order radiation below 11.6 eV, a lithium fluoride (LiF) filter was employed. The ionization region is equipped with ion optics consisting of one planar repeller and three extractor lenses, positioned in front of an octupolar RF‐driven ion guide. An effusive beam of the neutral precursor methanol (CH_3_OH/CD_3_OH/CD_3_OD) at a constant pressure of approximately 10^−^^6^–10^−5^ mbar is introduced through a needle valve located at the center of the ionization region, which is intersected at 90° by the photon beam. The resulting ions are extracted and transported into the octupole by the ion optics, oriented perpendicular to the photon beam propagation axis.

Neutral reagent (SO_2_) was subsequently introduced into the octupole cell via a leak valve, which finely regulates the pressure in the range of 10^−5^–10^−4^ mbar. The octupole functions as a two‐dimensional ion trap, providing an extended reaction region and efficient collection of ionic products formed in ion–molecule reactions. At the exit of the octupole, a quadrupolar deflector guides the ions into a quadrupole mass spectrometer equipped with a channeltron multiplier detector. The collision energy (CE) is determined by measuring the CH_3_OH^+^ yield as a function of the applied retarding field. An energy spread of approximately 110 meV was estimated.

Mass spectra were collected before and after the reaction of methanol radical cations with sulfur dioxide at a photon energy of 11.3 eV, covering the mass‐to‐charge range 10 <*m/z *< 100 and 25 <*m/z*< 85. Each spectrum was acquired over 10 or 30 s at a nominal SO_2_ pressure of about 8.0 × 10^−^^5^ mbar. The reactivity was studied as a function of photon energy by monitoring the intensities of reactant and product ions across the photon energy range 10.85 < hν < 11.60 eV, with steps of 0.05 eV and an acquisition time of 30 s per point, at sulfur dioxide pressures of 4.3, 8.3, and 11 × 10^−^^5^ mbar.

Methanol (CH_3_OH, HPLC gradient grade, 99.93%), CD_3_OH (99 atom % D), and CD_3_OD (99.8 atom % D) were purchased from Sigma‐Aldrich and used without further purification. Sulfur dioxide was purchased from Sigma–Aldrich with a purity > 99.98%.

### 2.2 Computational Details

The full geometry optimization of the products, reagents, and bimolecular adducts, as well as the Transition States (TS) and the harmonic vibrational analysis, were performed with the double‐hybrid density functional B2PLYP that incorporates a fraction of exact Hartree–Fock exchange and semilocal DFT exchange–correlation (Becke 88 + LYP) [28] using the Gaussian09 software [29]. The basis set used in this work is cc‐pVTZ, which is Dunning's correlation consistent of basis set at triple zeta [30]. For the reaction SO_2_ + CH_3_OH^+^ → HSO_2_ + CH_2_OH^+^, this level of calculation gives a reaction enthalpy of 93.6 kJ/mol at 298.15 K, which is about a 3.4% error with respect to the value of 96.9 kJ/mol obtained from data from NIST [31] formation enthalpies of CH_3_OH^+^, SO_2_, and CH_2_OH^+^ and the theoretical formation enthalpy of HSO_2_ [32]. The charge population was calculated following the Mulliken population analysis [33].

## 3 Results and Discussion

As reported in our previous works performed at the synchrotron ELETTRA [18, 19, 20], the methanol radical cation is produced by ionizing methanol in the ion source at a pressure of approximately 10^−^^5^ mbar, using tunable synchrotron radiation at the photon energy in the range from 10.8 to 11.6 eV. The ionization energy of methanol is 10.84 ± 0.01 eV [31], and to prevent the fragmentation of CH_3_OH^+^ into CH_2_OH^+^, which occurs at 11.649 ± 0.003 eV [34] we used the maximum hν at 11.6 eV. The possible formation of CH_2_O(H)H^+^, the most stable isomer of CH_3_OH^+^, by about 29.3–46.0 kJ/mol [35], can be excluded since the activation barrier is very high, 104.6 kJ/mol, not accessible in our experimental conditions [36]. In the first experiment, at a fixed photon energy of 11.3 eV, the mass spectrum of methanol radical cation was acquired to verify the presence of only methanol in the apparatus (signal at 32^+^), with only minor traces of water, which is protonated to form H_3_O^+^ (19^+^) by methanol ions (Figure 1a).

**FIGURE 1:**
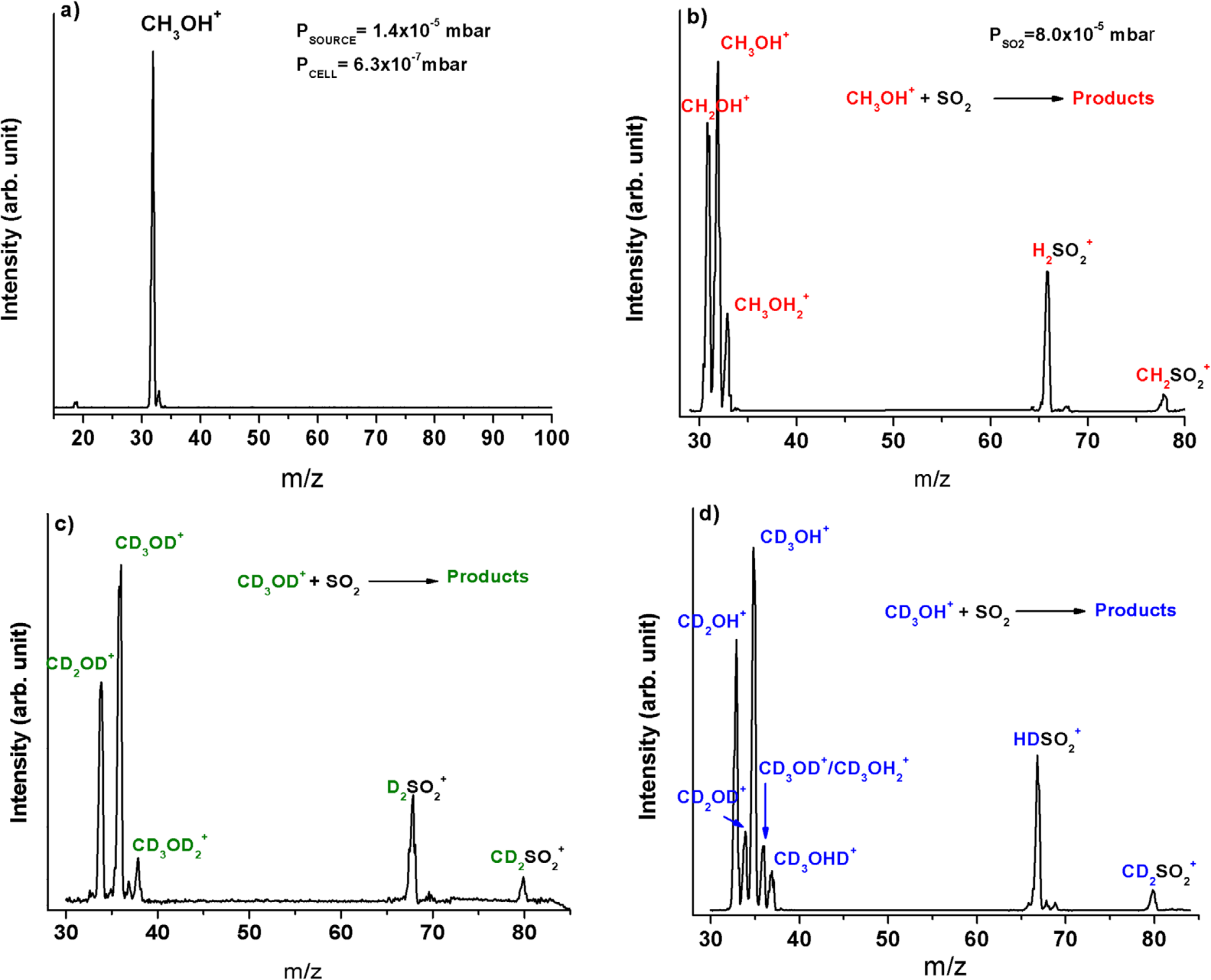
Mass spectra of the CH_3_OH, acquired at the photon energy of 11.3 eV without (a) and with (b) SO_2_ gas in the reaction cell at the pressure of 8.0 × 10^−5^ mbar. Reactivity of CD_3_OD^+^ (c) and CD_3_OH^+^ (d) with SO_2_ at a pressure of 8.0 × 10^−5^ mbar.

Subsequently, in a second experiment, SO_2_ was introduced into the reaction cell at a pressure of approximately 8.0 × 10^−^^5^ mbar, and the product ions, obtained from the ion‐molecule reaction, were detected using the quadrupole mass analyzer. In Figure 1b the mass spectrum of this experiment is shown, where the ions assigned to each peak are reported: CH_3_OH^+^ (molecular ion; *m/z* = 32), CH_2_OH^+^ (*m/z* = 31), CH_3_OH_2_^+^ (*m/z* = 33), H_2_SO_2_^+^ (*m/z* = 66) e CH_2_SO_2_^+^ (*m/z* = 78). The same experiment has been performed using CD_3_OD and CD_3_OH to better understand the chemistry of the reagents. The mass spectra registered are shown in Figure 1c,d. The data from Figure 1c confirms the products observed in the experiment with CH_3_OH: CD_3_OD^+^ (molecular ion; *m/z* = 36), CD_2_OD^+^ (*m/z* = 34), CD_3_OD_2_^+^ (*m/z* = 38), D_2_SO_2_^+^ (*m/z* = 68) e CD_2_SO_2_^+^ (*m/z* = 80). The data from Figure 1d clarify the role of hydrogen atoms of methanol in the ion chemistry: CD_3_OH^+^ (molecular ion; *m/z* = 35), CD_2_OH^+^ (*m/z* = 33), CD_3_OHD^+^ (*m/z* = 37), HDSO_2_^+^ (*m/z* = 67), CD_2_SO_2_^+^ (*m/z* = 80). Indeed, the signal at *m/z* = 67 (HDSO_2_^+^) and at *m/z* = 80 (CD_2_SO_2_^+^), without other signals around, demonstrates the role of both hydrogens, bound to C and O in CH_3_OH, involved in the formation of H_2_SO_2_^+^ and the nature of hydrogens in CH_2_SO_2_^+^. As regards the peaks observed in Figure 1c at *m/z* = 34 (CD_2_OD^+^), *m/z* = 36 (CD_3_OD^+^/CD_3_OH_2_^+^), they are due to H/D scrambling and are difficult to analyze. The formation of CH_3_O^+^/CD_3_O^+^ (*m/z* = 31, 34) has been excluded because the reaction of CH_3_OH^+^ + SO_2_ leading to HOSO and CH_3_O^+^ is endothermic by 236.4 kJ/mol [31, 32]. The unexpected formation of H_2_SO_2_^+^ and CH_2_SO_2_^+^ suggests a more complex reaction mechanism than a simple hydrogen atom or proton transfer between CH_3_OH^+^ and SO_2,_ as we have observed in our previous works [18, 19, 20]. To rationalize the entire process and identify the routes and the nature of all product ions, we suggest three initial reactions (Scheme 1) leading to CH_2_OH^+^, CH_2_OSO^+^, HOSOH^+^, ions, and HOSO, H_2_O, CH_2_O neutrals, respectively. We evaluated the thermochemistry for reactions (1) and (2) from databases and the literature [31, 37] and theoretically from our calculations (Table 1), considering the more stable isomers of H_2_SO_2_^+^, CH_2_SO_2_^+^ and HSO_2_, specifically: the HOSOH^+^ ion of sulphoxylic acid, the H_2_COSO^+^ ion and the hydroxysulfinyl radical HOSO [38]. The other neutrals are water and formaldehyde.

**SCHEME 1:**
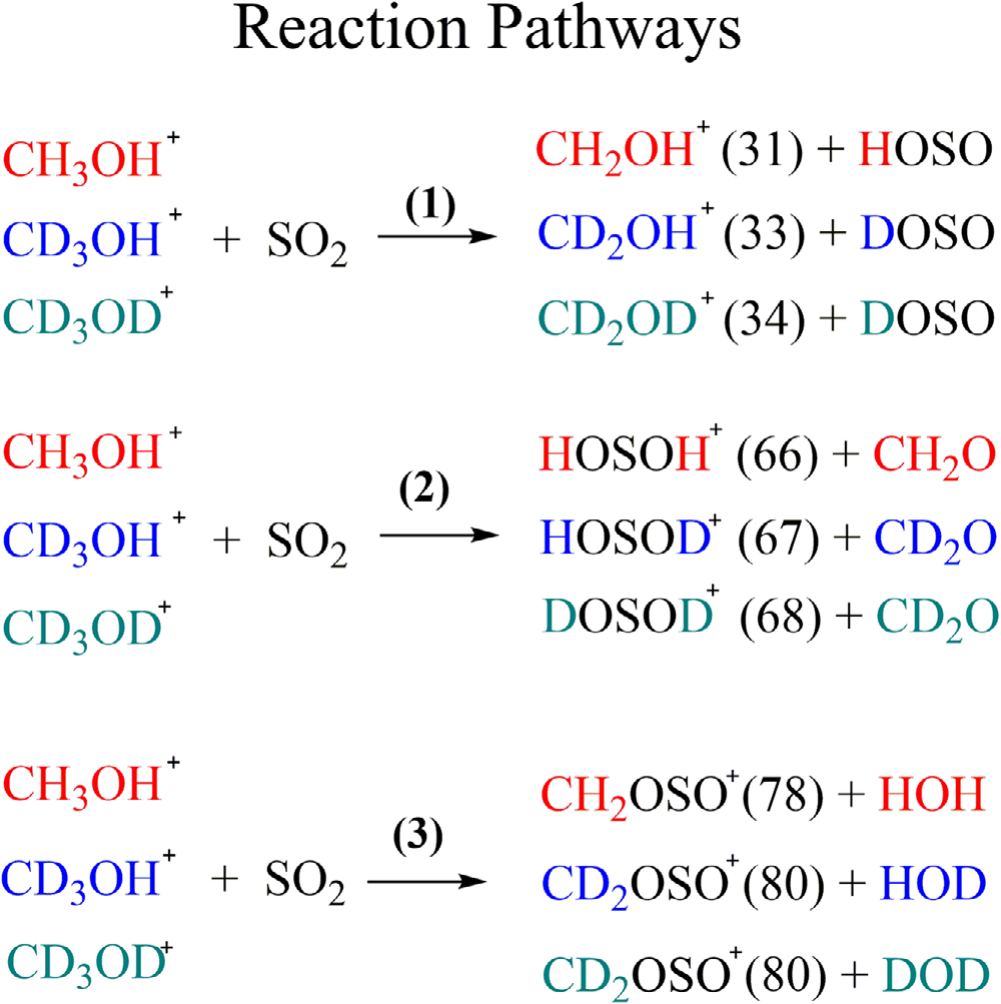
Three (1,2,3) reaction pathways between CH_3_OH^+^/CD_3_OH^+^/ CD_3_OD^+^ and SO_2_.

**TABLE 1:** Enthalpy (Δ*H*°_r_) from literature data and Energy (Δ*E*°_r_) of the three reactions calculated at the B2PLYP/cc‐pVTZ level of theory with ZPE.

Reaction	Δ*H*°_r_ ^a^	Δ*E*°_r_ ^a^
(1)	−96.9	−93.6
(2)	−110.7	−102.8
(3)	/	−114.4

^a^ kJ/mol.

The results show the high exothermicity of all reactions driven by the high stability of the products. The peak at *m/z* = 33 (CH_3_OH_2_^+^) in Figure 1b is probably due to a secondary reaction between HOSO neural and CH_3_OH^+^ since the process is highly exothermic, as the theoretical reaction enthalpy has been calculated as −280.2 kJ/mol. The complex reactivity of the system [CH_3_OH–SO_2_]^+^ has also been studied theoretically by *ab initio* calculations to understand the dynamics of these relevant processes. The energy profile and structures of minima and transition states calculated for the three pathways shown in Scheme 1 are reported in Figures 2 and 3.

**FIGURE 2:**
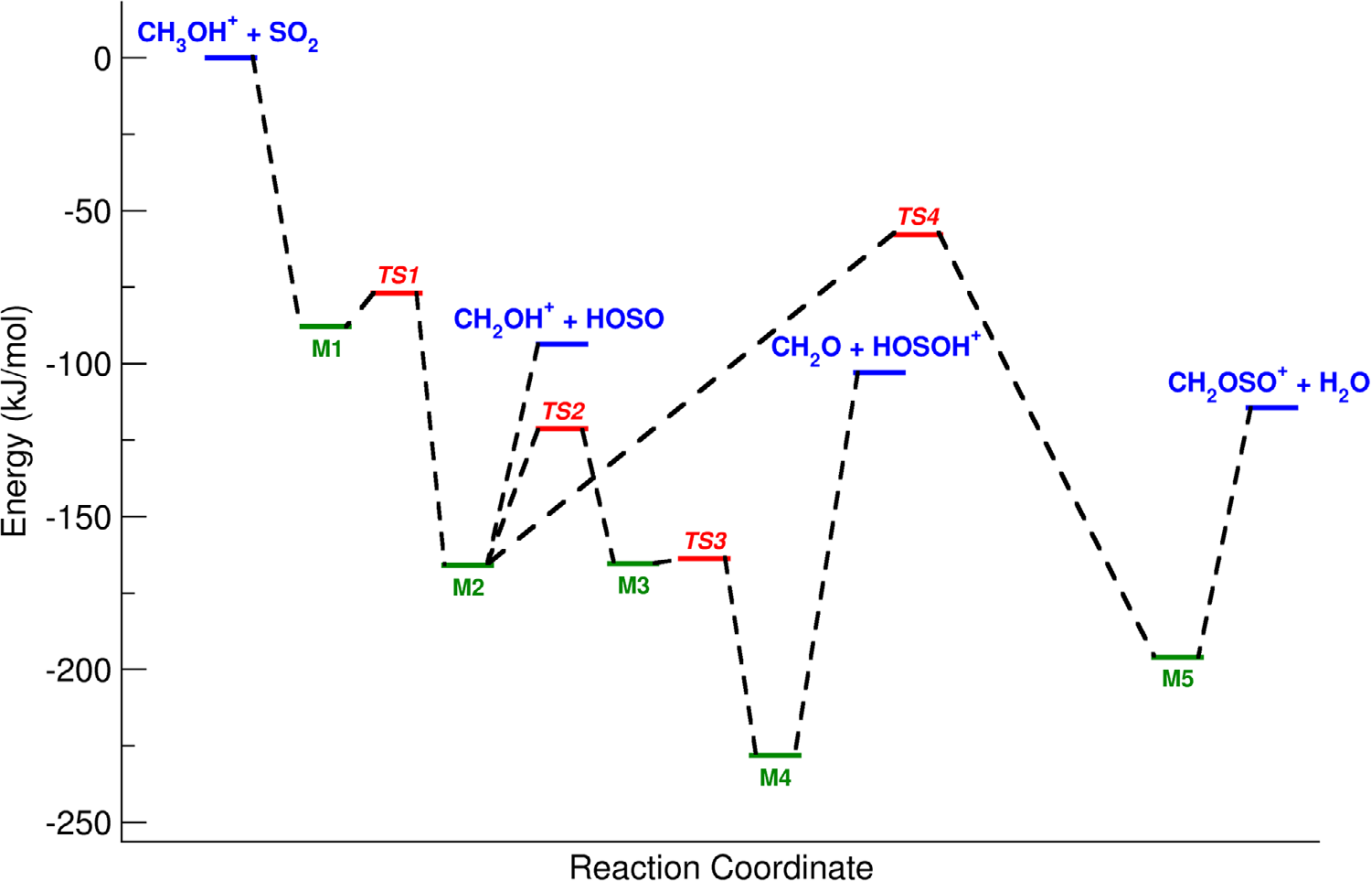
Potential energy profile (in kJ/mol including zero‐point energy) calculated at the B2PLYP/cc‐pVTZ level of theory.

**FIGURE 3:**
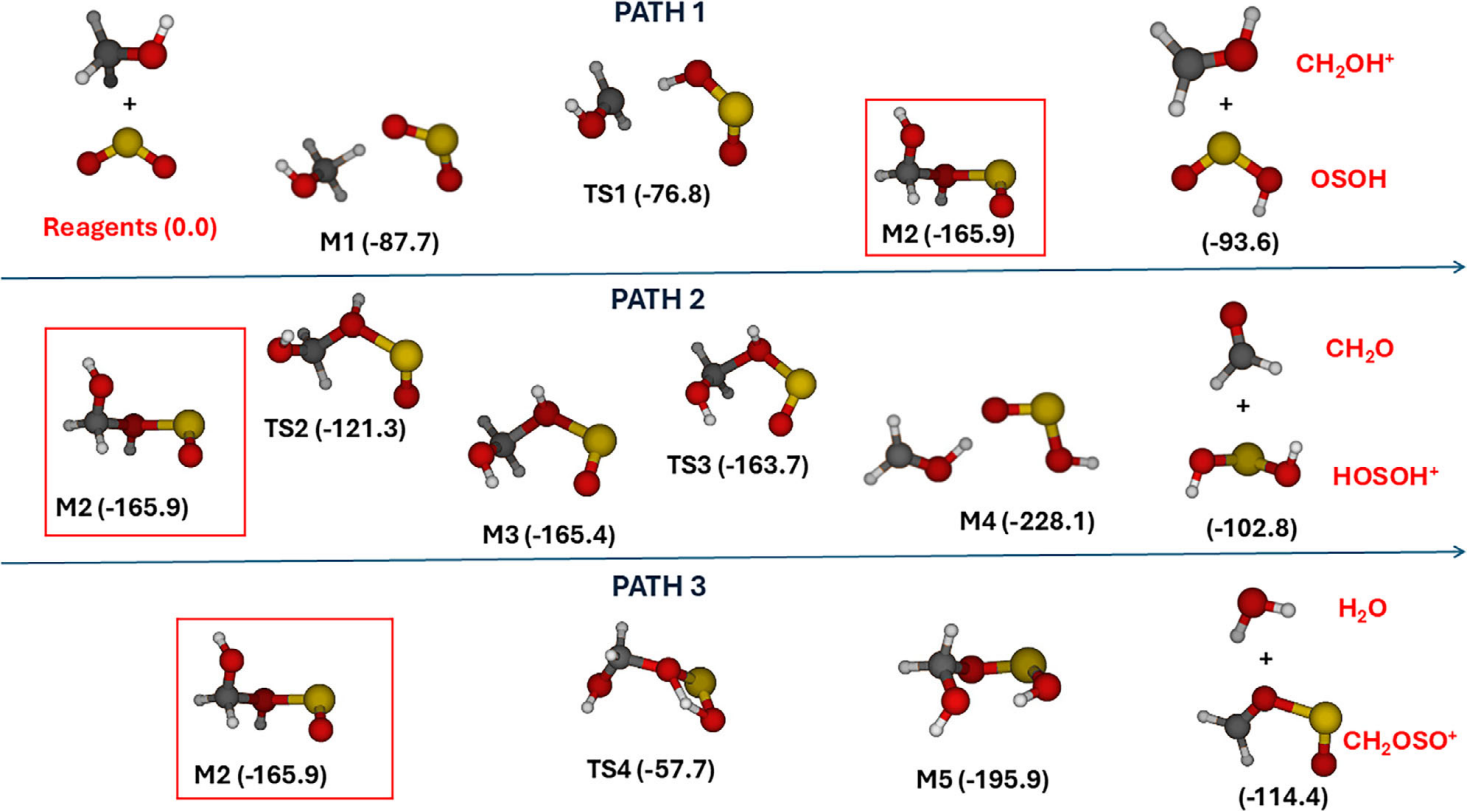
Optimized geometries and energies (kJ/mol) of the three paths calculated at the B2PLYP/cc‐pVTZ level of theory. The energy of both the reactants (fixed at 0) and the three sets of products is given as the sum of the energy of the two reactants and the three sets of the two products placed infinitely far apart.

The reaction of CH_3_OH^+^ and SO_2_ leads to an ion‐molecule encounter complex **M1** [HOCH_2_ **H**—OSO]^+^ at −87.7 kJ/mol with respect to reactants, and where one of the H, indicated in bold, of CH_3_ interacts with one of the O of OSO (Figure 3). This complex evolves through **TS1**, with a barrier of 10.9 kJ/mol, to minimum **M2** [HOCH_2_O(**H**)SO]^+^ at ‐165.9 kJ/mol, where the oxygen of OSO that interacted with the hydrogen **H** of the CH_3_ moiety of CH_3_OH, is now bound to **H** and a carbon atom (Figure 3). This bounded species, due to the exothermicity of the process, has an excess of internal energy and can follow three different pathways. The first one (path 1 in Figure 3) leads directly, without a TS, into CH_2_OH^+^, observed in the experiment, and HOSO. In the second path (path 2 in Figure 3), **M2** isomerizes into a rotational conformer **M3** at −165.4 kJ/mol through a barrier of 44.6 kJ/mol (**TS2**). In **M3** the ‐OH moiety of methanol points to the O atom of SO_2_ not linked to **H**. **M3** with a very low barrier of 1.7 kJ/mol (**TS3**) evolves into **M4** at −228.1 kJ/mol where the H of OH moiety in CH_2_OH are now strictly interacting with the O atom of SO_2_ not linked to **H** and the bond C─O in C─O(**H**)SO is broken. **M4** leads to the final products CH_2_O and **H**OSOH^+^. In the third path (path 3 in Figure 3), **M2** evolves into **M5** at −195.9 kJ/mol through a high barrier (**TS4**) of 108.2 kJ/mol. In **M5**, the **H** is now linked to the second O of OSO [HO‐CH_2_OSO**H**]^+^. Then, the **H** migrates into the OH moiety and the O─C bond in H(**H**)O‐CH_2_OSO breaks, leading to H_2_O and CH_2_OSO^+^. The charge distribution of the ionic reagents and products is shown in Figure 4, while the geometries of all structures are reported in the Supporting Information (Table S1).

**FIGURE 4:**
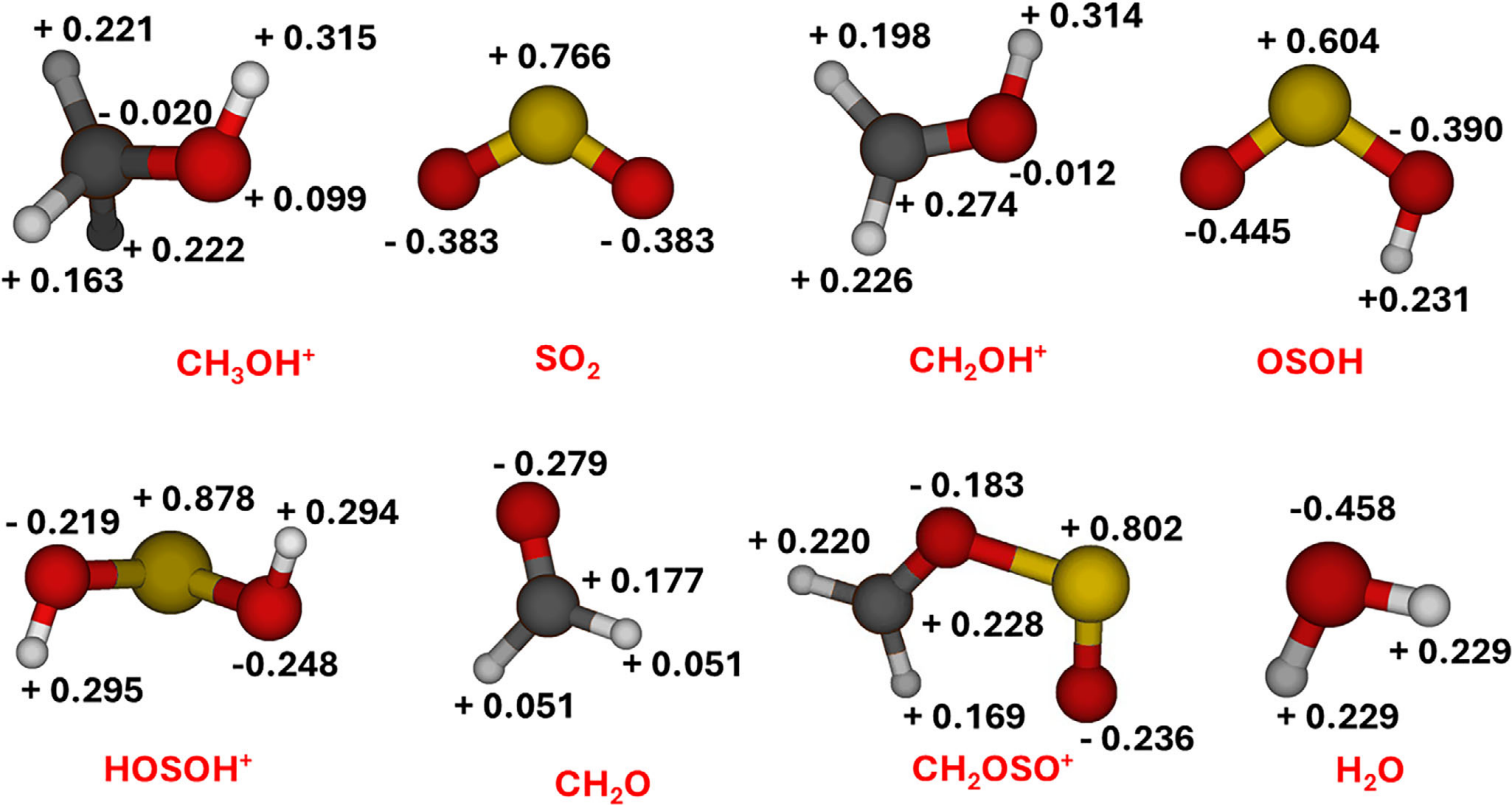
Mulliken Charge Population of the reagents and products.

The chemical species observed in this work play a significant role in various environments as discussed below. Formaldehyde (CH_2_O) is a key compound in both terrestrial atmospheric chemistry and astrochemical environments. In Earth's atmosphere, it represents a major intermediate in the oxidation of volatile organic compounds, particularly methane and other biogenic and anthropogenic hydrocarbons [39]. Its photolysis and reaction with OH radicals constitute primary sources of HOx radicals (OH and HOO), thus playing a crucial role in the atmospheric oxidative capacity and in the production of tropospheric ozone under NOx‐rich conditions [40]. In astrochemical environments, formaldehyde is one of the simplest yet most significant organic molecules from a prebiotic perspective. It has been observed in molecular clouds, comets, star‐forming regions, and protoplanetary disks through rotational and infrared spectroscopy [41]. Its formation can occur on icy dust grains via hydrogenation of CO followed by UV or cosmic‐ray irradiation [42]. These characteristics highlight formaldehyde as a benchmark species for understanding oxidative atmospheric processes on Earth and for elucidating the chemical evolution leading to the emergence of biologically relevant molecules in interstellar environments.

The formation of the sulfoxylic acid cation (HOSOH^+^) and hydroxysulfinyl radical (HOSO) is also of particular significance. Although they have not yet been directly detected in planetary atmospheres or in the interstellar medium, theoretical and experimental studies suggest that they may act as a pivotal intermediate in aerosol formation and in the sulfur chemistry of bodies such as Europa, Venus, and Io. Small sulfur oxoacids (H_x_SO_y_ with *x*, *y*  =  1–2) are recognized as highly reactive species in organic synthesis, biochemical pathways, and atmospheric processes, and are likely to contribute substantially to the chemistry of sulfur‐rich planetary environments and the interstellar medium [43]. CH_2_OH^+^ is the protonated formaldehyde, and CH_2_OSO^+^ is a very interesting, elusive species where the charge is distributed between CH_2_ and OSO moieties. This elusive species may contribute to the molecular growth of organic and sulfur compounds by serving as a source of “CH_2_^+^” and “SO^+^” moieties [44]. SO^+^ has been unambiguously detected in space [45], while CH_2_^+^ has not yet been observed. Nevertheless, its existence as an elusive intermediate is theoretically predicted, and it is believed to play a key role in molecular growth processes in astrochemical environments [46]. The third pathway may also operate when other linear alcohols react with SO_2_, since RCH_2_O─H species have C─H bond dissociation energies (BDE) lower than those of O─H bonds [47]. Consequently, RCH_2_OSO^+^ could be formed, potentially influencing both carbon and sulfur organic chemistry.

The reactivity has also been experimentally studied in the photon energy range 10.85 < hv < 11.6 eV, and the branching ratio (BR) of the products is reported in Figure 5.

**FIGURE 5:**
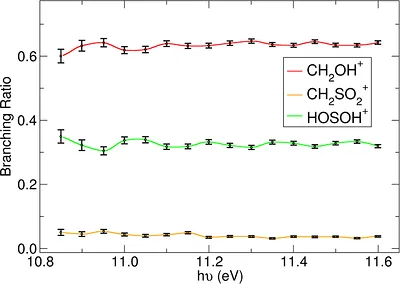
Branching ratios of the observed products as a function of photon energy.

These experimental BRs corresponding to the three reaction paths depicted in Scheme 1 can be interpreted considering the corresponding energy profiles shown in Figure 2. Notably, the path leading to the formation of CH_2_OSO^+^ is the most exothermic; however, it is also the least populated channel from an experimental standpoint. A second key observation concerns the formation of CH_2_OH^+^ and HOSOH^+^. Although these two products are associated with comparable exothermicities, their measured branching ratios differ significantly, with CH_2_OH^+^ being considerably more abundant than HOSOH^+^. This discrepancy clearly indicates that factors beyond simple thermodynamic considerations, specifically dynamical effects, play a decisive role in determining product distributions.

The relatively low yield of CH_2_OSO^+^ can be rationalized by considering the presence of **TS4**, which constitutes the rate‐limiting bottleneck along this path. Indeed, this route involves traversal of the highest‐energy transition state among the three considered mechanisms, thereby kinetically hindering product formation despite its favorable thermodynamics.

In contrast, this type of argument cannot account for the different branching ratios observed for CH_2_OH^+^ and HOSOH^+^. For these two channels, the highest energy points along their respective pathways correspond approximately to the product energies themselves, which are similar. Therefore, the observed disparity must arise from differences in how the available energy is partitioned during the reaction. Specifically, the formation of CH_2_OH^+^, which proceeds directly from intermediate **M2**, likely preserves a substantial fraction of the available reactive energy within the vibrational degrees of freedom of the system. Conversely, the pathway leading to HOSOH^+^ involves a more complex sequence, passing through two transition states (**TS2** and **TS3**) and two intermediates (**M3** and **M4**). This extended mechanism facilitates a redistribution of energy toward the reaction coordinate, at the expense of internal vibrational excitation. Consequently, the system evolving toward HOSOH^+^ possesses lower internal vibrational energy, resulting in a reduced density of accessible vibrational states and, therefore, a slower reaction rate. In other words, this channel proceeds more slowly but yields products with a comparatively higher fraction of translational (kinetic) energy. The BR data of Figure 5 demonstrate that path 1 is predominant and that all BR remain constant at all photon energies. In a gas‐phase ion–molecule reaction, if the branching ratios do not change when we vary the energy used to generate the ion, this suggests that the competing reaction pathways are likely governed to a comparable extent by the internal energy of the ion. This behavior can also be rationalized by considering that only a limited fraction of the photon energy (hν) is typically transferred to the ion during the photoionization process. In particular, for the case of methanol photoionization, we have evaluated the partitioning of hν between the kinetic energy of the emitted photoelectron and the internal energy of the resulting ion. The results, reported in Figure S1, clearly show that at an excess photon energy over the IE of ∼0.7 eV (hν = 11.6 eV), less than half of the available photoionization energy is retained by the ion. Specifically, only approximately 0.25 eV is converted into internal energy of the ion, with the remaining portion carried away as kinetic energy by the photoelectron. Therefore, it can be reasonably assumed that this excess internal energy of the ion (∼0.25 eV, corresponding to ∼24 kJ mol^−^^1^) does not significantly influence the relative formation rates of the three observed products. Indeed, in the context of the substantial amounts of reactive energy involved in this system (over 100 kJ/mol), the additional internal energy of the ion represents only a minor contribution and is unlikely to appreciably modify the relative kinetics of the competing pathways. Since all transition states lie at energies lower than that of the reactants, each pathway is effectively barrierless. Consequently, the reactions can proceed without an activation barrier, and the ion can react with the neutral molecule as soon as they approach each other, mainly due to long‐range electrostatic interactions such as ion–dipole or ion‐induced dipole forces. This behavior is typically described by capture models, such as the Langevin model [48]. We then calculated the rate coefficients for the three paths as a simple product of the total long‐range rate (with polarizability and dipole moment of SO_2_ of 26.20 bohr^3^ and 1.633 D, respectively) and the BR of each path: 0.60, 0.35 and 0.05 for path 1, 2, and 3, respectively, as reported in Figure 6. We assume that the BRs, which we measured at *T* = 300 K, remain constant as the temperature decreases. This assumption is justified by the high energy availability of all three reactive pathways since all TSs lie well below the energy level of the reactants and hence the contribution of thermal energy is negligible compared to the reaction energy. Therefore, the BRs are expected to be temperature independent below 300 K.

**FIGURE 6:**
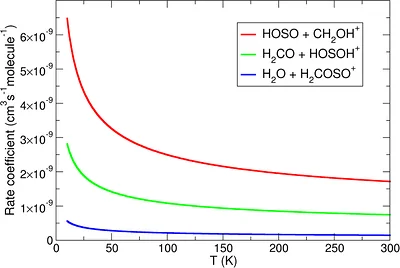
Rate coefficients as a function of temperature for the primary products. See the main text for the mixed experimental–theoretical procedure adopted to obtain these kinetic data.

We have also analyzed the formation pathway of CH_3_OH_2_^+^ from the possible secondary reaction between HOSO and CH_3_OH^+^. The geometric structures and the energy profile are shown in Figure 7a. It is clear that this reaction is also exothermic (−280.2 kJ/mol) and does not present a barrier. In fact, the TS is −269.7 kJ/mol lower in energy than the reactants. During the reaction, two intermediates, N1 and N2, are formed with energies of −304.2 and −370.1 kJ/mol, respectively. The N1 intermediate is characterized by an interaction between the oxygen of methanol and the sulfur of SO_2_. The TS is instead characterized by an interaction between the hydrogen of HOSO and the oxygen of methanol. Finally, the N2 intermediate presents a hydrogen bond between a hydrogen of the OH_2_ group of methanol and one of the two oxygen atoms of SO_2_. In Figure 7b, we report the long‐range rate coefficient (with polarizability and dipole moment of HOSO 24.47 bohr^3^ and 1.0659 D, respectively) of this secondary reaction between 10 and 300 K.

**FIGURE 7:**
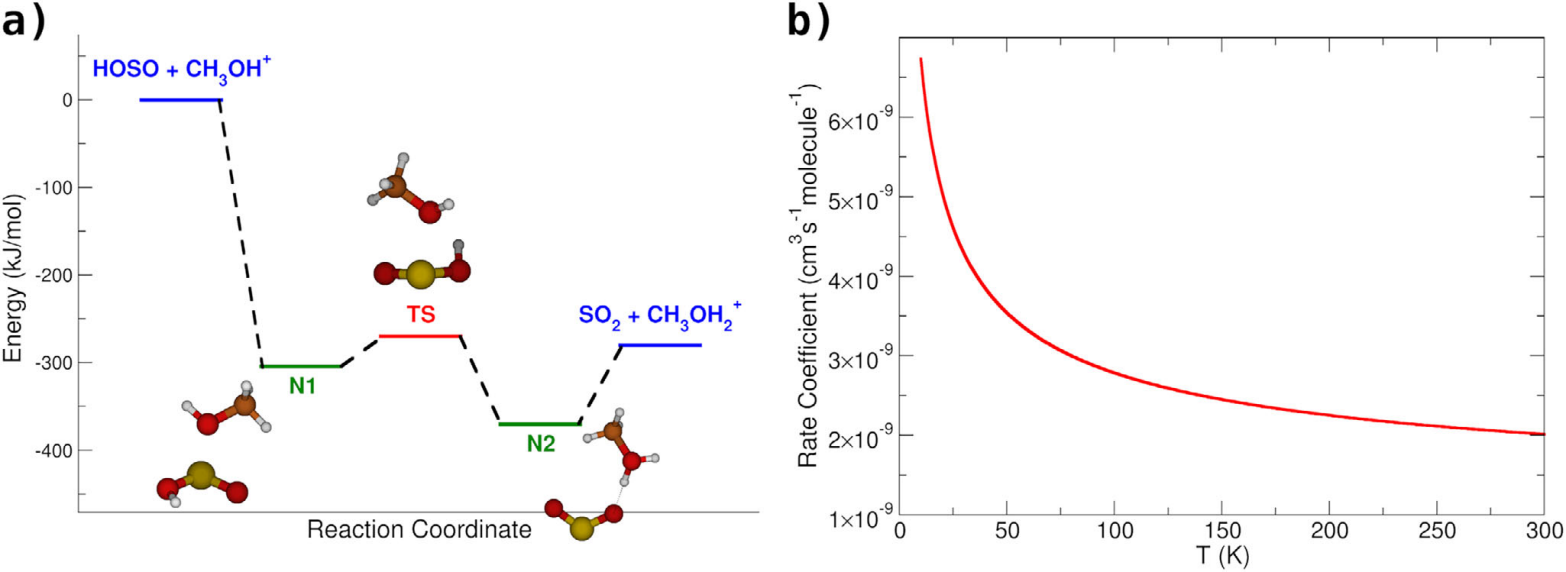
Secondary reaction: HOSO + CH_3_OH^+^ → SO_2_ + CH_3_OH_2_^+^. (a) Optimized geometries and energy levels; (b) Capture theory rate coefficient versus T.

## 4 Conclusions

In this work, we have demonstrated how an ionized mixture of CH_3_OH and SO_2_, present both in the atmosphere and in space, can give rise to complex chemistry, producing neutral and ionic species of great interest for both atmospheric chemistry and astrochemistry. The formation of these species occurs rapidly through three pathways, in which the initial [HOCH_2_O(**H**)SO]^+^ bound adduct is the key intermediate. The reaction extends beyond simple hydrogen or proton transfer reactions and gives a contribution to the awareness that a molecular mixture of SO_2_ and methanol in ionized environments can produce species such as H_2_O, CH_2_O, HOSO, CH_2_OH^+/^°, HOSOH^+/^°, CH_3_OH_2_^+/^°, and CH_2_OSO^+/^° ions and neutrals after rapid neutralization. Formaldehyde (CH_2_O) plays a central role in both atmospheric and astrochemical chemistry, acting as a key intermediate in oxidation processes on Earth and as a fundamental precursor of complex organic molecules in space. Its widespread presence in planetary and interstellar environments highlights its importance for understanding oxidative capacity and prebiotic chemical evolution. The formation of sulfur‐containing intermediates such as HOSOH^+^, HOSO, and CH_2_OSO^+^ suggests additional pathways linking carbon and sulfur chemistry. In particular, CH_2_OSO^+^ may act as a transient precursor of SO^+^ and CH_2_^+^ fragments, potentially contributing to molecular growth processes. Similar mechanisms may also occur in reactions involving other linear alcohols with SO_2_, further influencing sulfur‐ and carbon‐based chemistry.

It is known that solid‐phase processes occurring on icy dust grains play a dominant role in sulfur chemistry in many astrophysical environments, particularly under the very low temperatures (10–20 K). In these conditions, photolysis and radiolysis of ice mantles can lead to the formation of complex S‐bearing species, including molecules containing more than five atoms, and are also relevant in planetary environments such as the surface of Io. Nevertheless, many sulfur‐bearing molecules have been detected up to now in different gas‐phase ISM environments (CS, HCS^+^, H_2_CS, HSCN, HNCS, H_2_S, SH, SO, SO_2_, SO^+^, OCS, NS, NS^+^, CCS, CCCS, CH_3_SH, CH_3_CH_2_SH). Hence, gas‐phase‐ion‐molecule reactions involving sulfur‐containing species remain a crucial component of astrochemical networks. They contribute to the chemical evolution of diffuse clouds, help establish the ionization balance of the medium, and provide reactive species that can subsequently aggregate onto grain surfaces, thereby feeding solid‐phase chemistry. In this context, the present work focuses on the gas‐phase reaction between CH_3_OH^+^ and SO_2_, providing mechanistic and kinetic information that is currently not available but required for a comprehensive astrochemical description. The obtained branching ratios and temperature‐dependent rate coefficients represent important inputs for astrochemical models, where gas‐phase processes are intrinsically coupled to subsequent grain‐surface chemistry. In conclusion, this work aims to highlight the ion chemistry of molecular mixtures and pave the way for future experimental investigations of methanol–SO_2_ clusters under ionizing conditions, in both the liquid and solid phases. The work also provided the reaction coefficients for chemical networks useful in atmospheric and astrochemical models.

## Author Contributions

**Mauro Satta**: performed theoretical calculations, writing – original draft, review and editing. **Daniele Catone and Mattea Carmen Castrovilli**: performed experiments and data analysis, review and editing. **Antonella Cartoni**: supervision, conceptualization, performed experiments and data curation, writing – original draft, review and editing.

## Conflicts of Interest

The authors declare no conflicts of interest.

## Supporting information




**Supporting File 1**: chem71375‐sup‐0001‐SuppMat.pdf
The authors have cited additional references within the Supporting Information [17, 49, 50, 51].

## Data Availability

There is Supporting Information for this article.
