# Supplementary material for: The Rich Chemistry of Ionized Methanol With Sulfur Dioxide: Pathways to Prebiotic and Elusive Species in Earth's Atmosphere and Space
Source: Chemistry. 2026 Jul 9;32(39):e71375. doi: 10.1002/chem.71375 (PMC13715458; doi:10.1002/chem.71375)
Supplement: Supplementary file 1 [file CHEM-32-e71375-s001.pdf]

Supporting Information

The Rich Chemistry of Ionized Methanol with Sulfur Dioxide: Pathways to Prebiotic and Elusive Species in Earth's Atmosphere and Space

Mauro Satta,[1] Daniele Catone,[2] Mattea Carmen Castrovilli,[3] Antonella Cartoni[4,3]**

1) Institute of nanostructured materials-CNR (CNR- ISMN) Department of Chemistry, Sapienza University of Rome, P. le Aldo Moro 5, Rome, 00185, Italy

2) Istituto di Struttura della Materia - CNR (CNR-ISM), Area della Ricerca di Tor Vergata, Via del Fosso del Cavaliere, 00133, Rome, Italy

3) Istituto di Struttura della Materia - CNR (CNR-ISM), Area della Ricerca di Roma 1, Via Salaria km29.300, 00015, Monterotondo, Italy

4) Department of Chemistry, Sapienza University of Rome, P. le Aldo Moro 5, Rome, 00185, Italy

1 Average Internal Energy Distribution upon Photoionization

Following the same procedure reported in parag. 2.1 of the SI of the work Satta et al. [1] we use the equations (1) and (2) and the experimental literature data on threshold photoelectron spectra (TPES) cross-sections σ_j for methanol [2,3,4] to obtain the average ion internal and electron kinetic energy distribution upon photoionization shown in Figure S1:

$$E^{int}(h\nu) = \frac{\Sigma_j \sigma_j(h\nu)(h\nu_j - IE)}{\Sigma_j \sigma_j(h\nu)} \quad (1)$$

$$^{el}E_{KIN}(h\nu) = \frac{\Sigma_j \sigma_j(h\nu)(h\nu - h\nu_j)}{\Sigma_j \sigma_j(h\nu)} \quad (2)$$

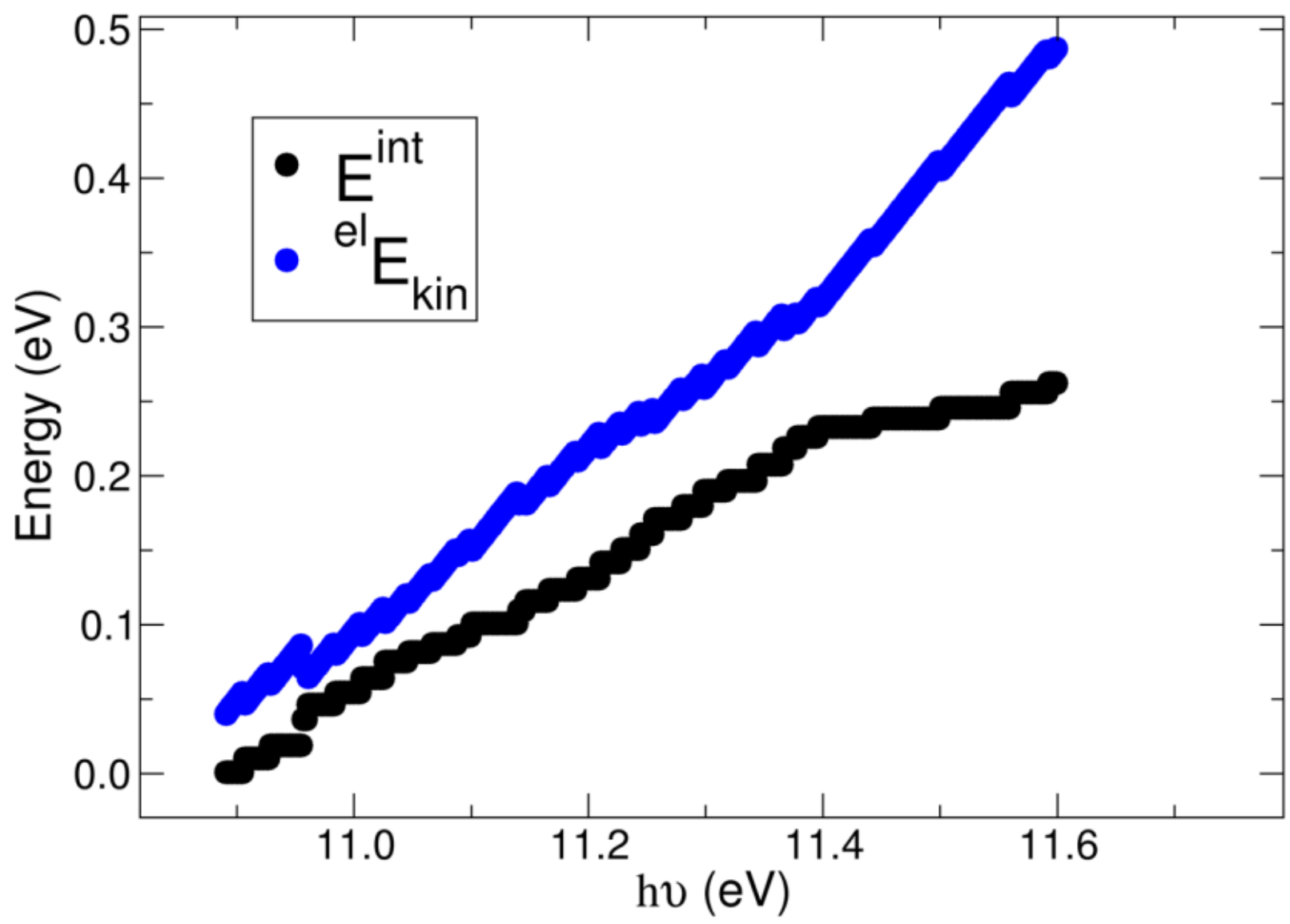

Fig. S1: Photoionization of MeOH. The kinetic energy of the photoemitted electron ($^{el}E_{kin}$) and the internal energy of the ionized MeOH (E^{int}) are reported as a function of the photoionization energy hν.

[1] Satta, M.; Cartoni, A.; Catone, D.; Castrovilli, M.C.; Bolognesi, P.; Zema, N.; Avaldi, L. The Reaction of Sulfur Dioxide Radical Cation with Hydrogen and its Relevance in Solar Geoengineering Models. Chem. Phys. Chem. 2020, 21, 1146–1156.

[2] Bethany Lowe, Alejandro L. Cardona, Maxi A. Burgos Paci, Paul M. Mayer, Probing the pyrolysis of methyl formate in the dilute gas phase by synchrotron radiation and theory, J Mass Spectrom. 2022;57:e4868.

[3] Tongpo Yu, Xiangkun Wu, Xiaoguo Zhou, Andras Bodi, Patrick Hemberger, Hydrogen migration as a potential driving force in the thermal decomposition of dimethoxymethane: New insights from

pyrolysis imaging photoelectron photoion coincidence spectroscopy and computations, Combustion and Flame 222 (2020) 123–132

[4] Zuyang Dai; Shuming Gao; Jia Wang; Yuxiang Mo Torsional energy levels of CH3OH+/CH3OD+/CD3OD+ studied by zero-kinetic energy photoelectron spectroscopy and theoretical calculations J. Chem. Phys. 141, 144306 (2014)

Table SI: Cartesian coordinates in Å for all the reagents, products, intermediates, and TS of the title reactions.

H_2O

O	0.000	0.000	0.118
H	0.000	0.757	-0.471
H	0.000	-0.757	-0.471

CH_2O

C	0.000	0.000	-0.529
O	0.000	0.000	0.675
H	0.000	0.935	-1.114
H	0.000	-0.935	-1.114

SO_2

S	0.000	0.000	0.372
O	0.000	1.251	-0.372
O	0.000	-1.251	-0.372

HOSO

S	0.000	0.506	0.000
O	1.307	-0.200	0.000
O	-1.209	-0.625	0.000
H	-0.781	-1.495	0.000

CH_3OH^+

C	-0.631	0.071	-0.001
O	0.704	-0.132	0.000
H	1.235	0.695	0.003
H	-1.056	-0.586	-0.811
H	-1.052	-0.573	0.824
H	-0.971	1.098	-0.005

CH_2OH^+

C	0.625	0.033	0.000
O	-0.612	-0.130	0.000
H	1.070	1.026	0.000
H	1.222	-0.874	0.000
H	-1.149	0.691	0.001

$HOSOH^+$

S	0.000	0.546	0.000
O	1.153	-0.544	0.000
O	-1.271	-0.362	0.000
H	-1.105	-1.328	0.000

H	2.051	-0.158	0.000

$CH_2SO_2^+$

S	0.000	0.990	0.000
O	1.386	0.543	0.000
O	-1.051	-0.679	0.000
C	-0.402	-1.729	0.000
H	-0.960	-2.664	0.000
H	0.693	-1.717	0.000

$CH_3OH_2^+$

C	-0.799	-0.002	0.000
O	0.705	-0.001	0.000
H	1.218	0.819	0.001
H	-1.103	-0.511	-0.905
H	-1.103	-0.507	0.907
H	-1.076	1.044	-0.002
H	1.214	-0.824	0.001

M1

C	-2.012	0.316	0.537
O	-2.749	0.097	-0.557
O	0.325	-0.944	0.152
S	1.650	-0.286	-0.083
O	1.652	1.156	-0.068
H	-3.285	-0.711	-0.489
H	-2.326	-0.177	1.456
H	-0.869	-0.254	0.337
H	-1.677	1.350	0.592

M2

C	1.551	0.052	0.551
O	0.346	1.008	0.055
S	-1.256	0.136	-0.441
O	-1.594	-0.775	0.645
O	1.843	-0.753	-0.461
H	2.463	-0.351	-1.084
H	2.286	0.802	0.829
H	0.156	1.711	0.705
H	1.121	-0.497	1.379

M3

C	-1.534	-0.243	0.500
O	-2.004	0.631	-0.373
O	-0.239	-1.037	-0.137
S	1.435	-0.252	-0.025
O	1.190	1.183	0.098
H	-1.594	1.503	-0.282
H	-2.192	-1.093	0.624
H	-0.447	-1.275	-1.062
H	-1.089	0.135	1.414

M4

C	-0.044	0.112	-0.015
O	0.094	-0.275	1.149
O	0.956	0.120	4.556
S	1.551	1.504	3.960
O	1.181	1.428	2.501
H	0.577	0.448	1.817
H	-0.516	-0.564	-0.722
H	1.150	0.039	5.506
H	0.299	1.099	-0.323

M5

C	1.403	-0.499	0.338
O	1.633	0.769	-0.130
O	0.121	-1.038	-0.270
S	-1.253	-0.324	-0.007
O	-0.987	1.228	0.104
H	2.154	0.764	-0.944
H	1.242	-0.473	1.410
H	-0.031	1.457	-0.041
H	2.128	-1.243	0.031

TS1

C	-0.096	0.101	-0.037
O	0.166	-0.270	1.216
O	2.421	0.214	-1.230
S	3.259	-1.046	-1.422
O	2.501	-2.264	-1.536
H	0.225	0.489	1.812
H	-0.436	1.113	-0.226
H	1.395	0.155	-0.838
H	-0.460	-0.713	-0.654

TS2

C	-1.422	0.376	0.440
O	-0.407	-0.759	0.348
S	1.418	-0.373	-0.237
O	1.664	1.019	0.096
O	-2.366	0.232	-0.523
H	-2.203	-0.496	-1.131
H	-1.834	0.287	1.443
H	-0.413	-1.309	1.151
H	-0.834	1.286	0.327

TS3

C	-1.552	-0.276	0.457
O	-0.125	-1.150	-0.088
S	1.400	-0.182	-0.034
O	0.946	1.203	0.138
O	-1.836	0.663	-0.401
H	-1.311	1.464	-0.241
H	-2.229	-1.118	0.422

H	-0.277	-1.494	-0.989
H	-1.151	-0.005	1.426

TS4

C	0.104	-0.082	-0.097
O	0.330	0.145	1.194
O	1.481	-0.444	-0.856
S	2.574	-1.589	-0.307
O	3.648	-0.512	-0.547
H	0.517	1.075	1.379
H	-0.201	0.755	-0.715
H	2.612	0.134	-0.874
H	-0.468	-0.987	-0.252

N1

O	-1.993	-0.543	-0.331
C	-2.514	0.739	0.220
H	-2.559	0.668	1.300
H	-3.488	0.884	-0.228
H	-1.813	1.493	-0.113
H	-2.535	-1.310	-0.092
H	-1.003	-0.715	-0.122
O	0.460	-1.010	0.134
S	1.684	-0.179	0.064
O	1.475	1.230	-0.189

N2

C	-2.273	0.141	0.094
O	-1.081	-0.720	0.051
S	0.750	0.179	-0.448
O	1.569	-0.944	0.280
O	0.611	1.363	0.352
H	-3.018	-0.376	0.690
H	-2.629	0.339	-0.912
H	-1.965	1.053	0.591
H	-1.259	-1.523	-0.463
H	1.710	-0.794	1.238

TS

C	2.945	0.204	-0.423
O	1.908	-0.090	0.537
O	-0.808	-1.204	-0.084
S	-1.799	-0.030	-0.077
O	-1.219	1.267	0.096
H	3.506	-0.691	-0.683
H	3.616	0.963	-0.028
H	2.455	0.595	-1.308
H	2.324	-0.386	1.352
H	0.166	-1.006	0.055
